# A dosimetric comparison of three‐dimensional conformal radiotherapy, volumetric‐modulated arc therapy, and dynamic conformal arc therapy in the treatment of non‐small cell lung cancer using stereotactic body radiotherapy

**DOI:** 10.1120/jacmp.v15i5.4898

**Published:** 2014-09-08

**Authors:** Bradley M. Rauschenbach, Luke Mackowiak, Harish K. Malhotra

**Affiliations:** ^1^ Department of Radiation Medicine Roswell Park Cancer Institute Buffalo NY USA

**Keywords:** SBRT, VMAT, DCAT, lung cancer

## Abstract

This study evaluates three‐dimensional conformal radiotherapy (3D CRT), volumetric‐ modulated arc therapy (VMAT), and dynamic conformal arc therapy (DCAT) planning techniques using dosimetric indices from Radiation Therapy Oncology Group (RTOG) protocols 0236, 0813, and 0915 for the treatment of early‐stage non‐small cell lung cancer (NSCLC) using stereotactic body radiotherapy (SBRT). Twenty‐five clinical patients, five per lung lobe, previously treated for NSCLC using 3D CRT SBRT under respective RTOG protocols were replanned with VMAT and DCAT techniques. All plans were compared using respective RTOG dosimetric indices. High‐ and low‐dose spillage improved for VMAT and DCAT plans, though only VMAT was able to improve dose to all organs at risk (OARs). DCAT was only able to provide a minimal improvement in dose to the heart and ipsilateral brachial plexus. Mean bilateral, contralateral, and $V_{20}$ (percentage of bilateral lung receiving at least 20 Gy dose) doses were reduced with VMAT in comparison with respective 3D CRT clinical plans. Though some of the DCAT plans had values for the above indices slightly higher than their respective 3D CRT plans, they still were able to meet the RTOG constraints. VMAT and DCAT were able to offer improved skin dose by 1.1% and 11%, respectively. Monitor units required for treatment delivery increased with VMAT by 41%, but decreased with DCAT by 26%. VMAT and DCAT provided improved dose distributions to the PTV, but only VMAT was consistently superior in sparing dose to OARs in all the five lobes. DCAT should still remain an alternative to 3D CRT in facilities that do not have VMAT or intensity‐modulated radiotherapy (IMRT) capabilities.

PACS numbers: 87.53.Ly, 87.55.dk, 87.55.D‐

## I. INTRODUCTION

Surgery remains the primary choice in the treatment of early‐stage non‐small cell lung cancer (NSCLC). However, not all patients are considered medically operable because of comorbidities, advanced age, or unwillingness to undergo surgery. These patients require a nonsurgical treatment and are primarily referred to radiation therapy for treatment. Unfortunately, standard radiation therapy regimens involving conventional fractionation schemes for such type tumors have delivered only mediocre results so far.^1^, ^2^ In recent times, a new form of extreme hypofractionation regimen is becoming popular, delivering a very high ablative radiation dose per fraction in 1 to 5 fractions. Commonly known as stereotactic body radiotherapy (SBRT), this alternative to conventional fractionation has produced excellent results in the treatment of medically inoperable NSCLC with local control rates of 95%.^1^, ^2^ Initial clinical results are showing a reduced local failure rate comparable to that of surgery in early‐stage NSCLC patients.^(3)^

SBRT is quickly becoming a standard treatment option over conventional fractionation for patients with medically inoperable early‐stage NSCLC. The areas of high dose conforming tightly to the PTV and dose rapidly falling off into surrounding tissues make SBRT an attractive treatment option.

Many institutions deliver SBRT using three‐dimensional conformal radiotherapy (3D CRT), involving the use of seven to eleven static, nonopposing, coplanar, and noncoplanar beams with approximately equal weightings. This popular method results in a highly conformal dose distribution surrounding the tumor in all directions. While SBRT treatments using 3D CRT have produced excellent treatment plans, beam arrangements for each patient must be customized. Treatments using 3D CRT result in lengthy delivery times because of varying gantry, collimator, and couch positions. A new generation of linear accelerators now supports radiation dose delivery using volumetric‐modulated arc therapy (VMAT) and dynamic conformal arc therapy (DCAT). VMAT utilizes a rotating gantry up to 360° with dynamic multileaf collimator (MLC) motion, variable dose rates, and gantry speed modulation.^(4)^ Like VMAT, dynamic conformal arc therapy (DCAT) also utilizes a rotating gantry up to 360° with MLC motion, but only shapes the MLCs to the target along the arc pathway.^(5)^ Both treatment techniques are capable of expediting the treatment delivery process significantly.

Shi et al.^(6)^ have successfully used modified DCAT technique for five patients (3 RTOG‐0236 & 2 RTOG‐0813) and were able to meet the respective protocols constraints. In 14 patients, Merrow et al.^(7)^ found VMAT treatment plans comparable to 3D CRT plans. In another study for nine patients, Kannarunimit et al.^(8)^ compared robotic radiosurgery, helical tomotherapy, and VMAT plans against their respective 3D CRT plans and found satisfactory plans with either of them. Our study systematically compares 3D CRT against DCAT, as well as VMAT, for lung tumors in all its five lobes, using standard dosimetric indices using the same respective patient CT dataset. The latter angle is important as the critical structures and their relative separation from tumors is different in different lobes of lung. The relative strengths and weakness of each technique is discussed in detail in the paper. Such a side‐by‐side comparison can provide strengths and weaknesses of either technique in an objective way.

Accordingly, this retrospective study investigates the potential dosimetric benefits of VMAT and DCAT with 3D CRT in the treatment of early‐stage NSCLC using indices for tumors and organs at risk (OARs) established by Radiation Therapy Oncology Group (RTOG) protocols 0236, 0813, and 0915^9^, ^10^, ^11^ and by our institutional review board approved protocol. For practical purposes, all 3D CRT plans used clinically were considered the reference standard against which VMAT/DCAT plans were intercompared.

## II. MATERIALS AND METHODS

### A. Patient selection

**Figure 1:**
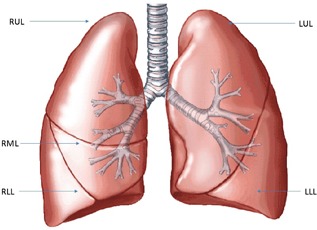
Schematic of a human lung with its five lobes. RUL=right upper lobe,RML=right middle lobe,RLL=right lower lobe,LUL=left upper lobe,LLL=left lower lobe.

Twenty‐five patients, five per lung lobe, with medically inoperable stage I/II NSCLC previously treated with 3D CRT SBRT were selected for this study (Table 1, Fig. 1). Patients represented various tumor sizes and fractionation schemes (Table 2). VMAT and DCAT plans were generated for each patient in Eclipse version 10.0 treatment planning software (Varian Medical Systems, Palo Alto, CA) using the analytic anisotropic algorithm (AAA) with a computation grid size of 2.5 mm. All treatment plans were evaluated using dosimetric indices from respective RTOG protocols.

The patients had received different prescription doses based on their respective clinical protocol and those doses were not changed in the subsequent VMAT or DCAT plans (Table 2). The fractionation scheme for RTOG 0236 was 60 Gy in 3 fractions (n = 13), 40 Gy in 5 fractions (n = 1) was the scheme for RTOG 0813, and RTOG 0915 contained a two‐arm scheme of either 34 Gy in 1 fraction (n = 1) or 48 Gy in 4 fractions (n = 3). Seven patients received 30 Gy in 1 fraction from an internal protocol at our institute (30 Gy in single fraction). The appropriate protocol guidelines were adhered to for all patients, even when not formally enrolled in study.

**Table 1:** Patient characteristics (n = 25)

Sex	
Male	12
Female	13
RTOG protocol	
0236	13
0813	1
0915	4
Other^a^	7
Volume ITV (cm^3^)	
Mean	12.60
Range	1.45 ‐ 85.78
Volume PTV (cm^3^)	
Mean	41.08
Range	12.65 ‐ 190.70

a Internal protocol 30 Gy in single fraction.

**Table 2:** Treatment plan conditions for all the 25 patients used in the study

*Case*	*ITV (cm* ^*3*^ *)*	*PTV (cm* ^*3*^ *)*	*Prescribed Dose (Gy)*	*Number of Fractions*	*Heterogeneity Corrections*	*RTOG Protocol*
1	4.84	23.97	60	3	No	0236
2	9.02	37.98	30	1	Yes	^a^
3	3.91	20.47	30	1	No	^a^
4	5.67	25.78	60	3	No	0236
5	4.92	28.58	60	3	No	0236
6	3.17	19.3	48	4	Yes	0915
7	8.65	34.22	60	3	No	0236
8	8.47	37.43	60	3	No	0236
9	36.78	92.79	60	3	No	0236
10	14.98	51.59	60	3	No	0236
11	3.01	17.54	60	3	No	0236
12	7.15	29.63	30	1	Yes	^a^
13	3.92	20.02	34	1	Yes	0915
14	9.52	39.77	60	3	No	0236
15	6.1	27.12	60	3	No	0236
16	4.3	22.94	48	4	Yes	0915
17	85.78	190.7	40	5	Yes	0813
18	7.7	35.98	30	1	No	^a^
19	6.24	29.5	30	1	No	^a^
20	1.45	12.65	60	3	No	0236
21	5.09	18.41	48	4	Yes	0915
22	15.05	50.03	30	1	Yes	^a^
23	8.27	36.03	30	1	No	^a^
24	20.89	62.51	60	3	No	0236
25	2.11	12.88	60	3	No	0236

a Internal protocol (RTOG 0236 vs. 30 Gy in single fraction).

### B. Treatment planning volumes

CT datasets were acquired using a GE LightSpeed 16 slice CT scanner (RT16) (GE Healthcare, Waukesha, WI). Four‐dimensional (4D) CT scans were captured for all patients during uncoached respiration using Varian's real‐time position management (RPM) system. A 1.25 mm slice thickness was used around the region of interest and all critical structures. An infrared block was placed on the patient's chest during image acquisition to track breathing motion during all phases of the respiratory cycle. Patients were immobilized in the supine position with the arms above the head in a full‐body vacuum bag, and abdominal compression plates were used to further immobilize all patients during image acquisition and treatment.

Retrospective CT images were grouped into ten bins, each reflecting 10% of the respiratory cycle, with 0% and 50% representing full inspiration and full expiration, respectively. The gross tumor volume (GTV) was contoured on each of the ten CT image sets. An internal target volume (ITV) was then created from the Boolean sum of the individual ten GTVs. The planning target volume (PTV) was created by expanding the ITV 0.5 cm in the axial plane and 0.5 cm in the cranial–caudal plane. The mean ITV volume was 12.60 cm^3^ (range $1.45\;‐\;85.78\;{\text{cm}}^{3}$), and the mean PTV volume was 41.08 cm^3^ (range $12.65\;‐\;190.70\;{\text{cm}}^{3}$) (Table 1).

Critical structures were contoured according to the guidelines provided by respective RTOG protocols. Structures used for plan comparison in this study included the ipsilateral brachial plexus, proximal bronchial tree, esophagus, heart, spinal cord, proximal trachea, and the lungs. The contralateral, ipsilateral, and bilateral lung structures were contoured as separate structures. The patient's skin, defined as the inner 0.5 cm of the body structure, was contoured for all patients to compare skin dose. Image sets and contours from 3D CRT plans were used in VMAT and DCAT plans to ensure a meaningful comparison.

### C. Planning techniques

At the time of CT acquisition, the isocenter was placed within the tumor volume at the approximate center of mass. A 3D CRT plan was generated using seven to eleven static, nonopposing, coplanar and noncoplanar beams. 6 MV photon beams were generally used. Occasionally a maximum of two beams of higher energy were used in accordance with RTOG protocols. MLCs were conformed to the PTV for each beam with no additional margin, except in superior–inferior direction where a margin of 1 mm was used wherever needed. The beam arrangement was customized for each patient based on tumor location and nearby OARs. Plans were normalized at the isocenter, and an isodose prescription line for PTV coverage was chosen such that at least 95% of the PTV received 100% of the prescription dose, and 99% of the PTV was covered by at least 90% of the prescription dose. Isodose prescription lines were required to fall between 60% and 90%, with most falling between 80% and 90% in this study. An expected area of high dose was found within the PTV equal to the reciprocal of the isodose prescription line chosen. Plans were manually optimized to meet all tumor and normal tissue objectives based on respective RTOG protocols. These 3D CRT plans were used clinically and were subsequently used for intercomparison with their respective VMAT/DCAT plans.

Initially, VMAT plans were designed with two ipsilateral, coplanar arcs (n = 8). If respective RTOG constraints could not be fulfilled, two additional contralateral, coplanar, posterior oblique arcs up to 45° (n = 9) were added in an attempt to reduce the amount of contralateral lung being irradiated. If constraints still could not be satisfied, two full arcs were used (n = 8). Full coplanar arcs covered 358°. Due to treatment machine limitations, arcs cannot pass through 180°, defined as the posterior–anterior direction. Four coplanar arc plans consisted of two ipsilateral arcs of 179° and two contralateral arcs up to 45°. Two coplanar arc plans consisted of two ipsilateral arcs of 179°. All clockwise arcs used a 30° collimator rotation, while counterclockwise arcs used a complementary 330° rotation. Collimator rotations were used to minimize the MLC tongue‐and‐groove effect for arc‐based plans. During the VMAT optimization process, PTV dose objectives were typically set 3% ‐ 7% higher than the prescribed dose. Once the PTV achieved the desired dose in the optimization process, dose to each OAR was manipulated using inverse optimization constraints until PTV coverage began to deteriorate. At that point, the OAR was no longer manipulated in the optimization process.

**Figure 2:**
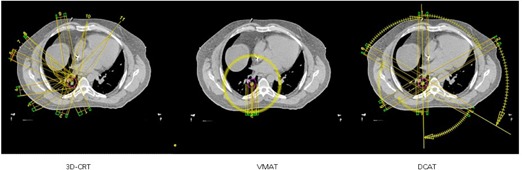
Comparisons in the beam arrangements between 3D CRT (left), VMAT (middle), and DCAT (right) for a typical case in a transverse plane passing through the center of the tumor. Also shown is the PTV volume.

DCAT plans were generated using one full arc (n = 13) or six subarcs (n = 12). Like VMAT, full arcs covered 358° due to machine limitations. MLCs were conformed to the PTV with no additional margin. For plans failing to meet respective RTOG objectives, single‐arc plans were replaced with six subarcs: four arcs of 60° and two arcs at 59°. A 30° collimator rotation was used for all DCAT plans. Any heterogeneity corrections used in 3D CRT plans was also used in VMAT and DCAT plans (Table 2). Figure 2 shows a typical arrangement of beams/arcs for 3D CRT and VMAT/DCAT for a representative patient. No VMAT or DCAT plans provided dramatically dissimilar results to clinical 3D CRT plans.

Similar to 3D CRT plans, both VMAT and DCAT plans were also normalized to the isocenter. To ensure an “apple‐to‐apple” comparison, the prescribed isodose line in VMAT and DCAT plans were suitably adjusted to ensure that at least 95% of the PTV volume is getting 100% of the prescription dose.

The planning process involved with VMAT and DCAT is more planner‐friendly compared to 3D CRT. The use of the Arc Geometry Tool in Eclipse treatment planning software for VMAT, or the use of field templates for DCAT, makes field placement a quick process. The inverse optimization process for VMAT plans happens to be the most time‐consuming aspect of VMAT planning. DCAT plans are calculated like conventional forward‐planning techniques in approximately 15 min. If subarcs are used with DCAT, field weighting can quickly be adjusted once plans are calculated to respect OAR constraints.

After plan completion, all 3D CRT plans were verified on the treatment machine for possible gantry‐couch collisions. The verification requires the planner to set up the vacuum bag and abdominal compression plates on the assigned treatment machine, and move the gantry and couch to each field position to verify proper clearance. If a collision does exist, the plan must be revised by the planner until a new, acceptable plan is achieved. The verification process takes about 15 min, which can be difficult to schedule on busy treatment machines during the day, and typically requires the collaboration with radiation therapists working on the treatment machine.

### D. Plan comparison

Treatment plans were compared based on PTV coverage, dose to OARs, and high‐ and low‐dose spillage guidelines established by respective RTOG protocols. Additionally, the plans were also compared for monitor units (MUs) required for treatment delivery.

#### D.1 PTV coverage

Plans were required to deliver 100% of the prescription dose to at least 95% of the PTV and at least 90% of the prescription dose to 99% of the PTV. For a meaningful comparison, all 3D CRT, VMAT, and DCAT plans satisfied this condition.

#### D.2 Organs at risk (OARs)

Plans were compared based on whether they were able to meet maximum point dose ($D_{\text{max}}$) constraints established by respective RTOG protocols. OARs included the ipsilateral brachial plexus, proximal bronchial tree, esophagus, heart, spinal cord, trachea, and skin. $D_{\text{max}}$ constraints for OARs established by respective RTOG protocols are found in Table 3. Mean doses to the bilateral and contralateral lung were recorded, though no dose constraints were established by respective RTOG protocols. The volume of the bilateral lung receiving at least 20 Gy $\left(V_{20}\right)$ should be ideally < 10% for all respective RTOG protocols, with an allowable minor deviation up to 15%. The ITV was subtracted out of all lung volumes.

**Table 3:** RTOG protocol dose limits to OARs (Gy)

	*RTOG 0236*	*RTOG 0813*	*RTOG 0915*	
	20 Gy × 3	8 Gy × 5	34 Gy × 1	12 Gy × 4
Ipsilateral Brachial Plexus	24	32	17.5	27.2
Ipsilateral Bronchial Tree	30	42	20.2	34.8
Esophagus	27	42	15.4	30
Heart	30	42	22	34
Spinal Cord	18	30	14	26
Proximal Trachea	30	42	20.2	34.8

#### D.3 Conformality index (CI)

The target CI is the ratio of the volume receiving the prescription dose to PTV volume, and is ideally < 1.2 for all respective RTOG protocols. An allowable minor deviation up to 1.4 was permitted.

#### D.4 *High‐dose location* (${\text{HD}}_{\text{loc}}$)

The volume of the tissue outside of the PTV receiving greater than 105% of the prescription dose must be less than 15% of the PTV volume.

#### D.5 *Low‐dose location* ($D_{2\text{cm}}$)

The maximum dose in normal tissue 2 cm from the PTV in all directions was defined for each plan according to PTV volume and respective RTOG protocol (RTOG‐0236, RTOG‐0813, RTOG‐0915).

#### D.6 *Low‐dose volume* ($R_{50\%}$)

The ratio of the volume receiving 50% of the prescription dose to PTV volume was defined for each plan according to PTV volume and respective RTOG protocol (RTOG −0236, RTOG‐0813, RTOG‐0915).

#### D.7 Monitor units (MUs)

The monitor units required for treatment delivery were recorded for each treatment planning technique.

The CI, ${\text{HD}}_{\text{loc}}$, $R_{50\%}$, and bilateral $V_{20}$ were compared by finding the mean average among all 25 cases. Due to varying prescription doses between respective RTOG protocols, $D_{2\text{cm}}$, $D_{\text{max}}$, and MUs could not be directly compared. Instead, the ratio of each to the prescription dose ($D_{P}$) found, resulting in $D_{2\text{cm}}/D_{P}$, $D_{\text{max}}/D_{P}$, and $\text{MU}/D_{P}$. These ratios were then compared by finding the mean average among all 25 cases.

## III. RESULTS

Dose‐volume histograms (DVHs) were generated for each planning technique and used to compare PTV coverage, dose to OARs, and high‐ and low‐dose spillage. Similar to 3D CRT, all VMAT and DCAT plans met respective RTOG protocol criteria for PTV coverage.

Comparison between treatment planning techniques is summarized in Table 4. All VMAT and DCAT plans either met respective RTOG normal tissue dose constraints or provided similar results to 3D CRT plans. Mean bilateral, contralateral, and $V_{20}$ doses for lung improved with VMAT by 13%, 4.2%, and 6.3%, respectively. All three lung parameters increased with DCAT, most notably the mean contralateral lung by 60%. Minimal increases of 0.3% and 3.4% were observed in the mean bilateral and $V_{20}$ lung doses, respectively. Only one case was unable to keep $V_{20}$ below 10% between all treatment planning techniques. This common case was able to satisfy the minor deviation of $V_{20}$ less than 15% in all treatment planning techniques. Doses to all OARs improved dramatically with VMAT. DCAT was only able to improve dose to the heart and ipsilateral brachial plexus by 3.4% and 2.8%, respectively. Skin dose improved with DCAT by 11% and by 1.1% with VMAT.

**Figure 3:**
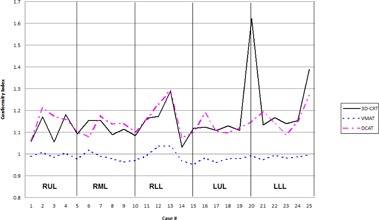
Comparison in the conformality index between 3D CRT, VMAT, and DCAT. The high value in Case 20 for 3D CRT is due to a small PTV volume

**Figure 4:**
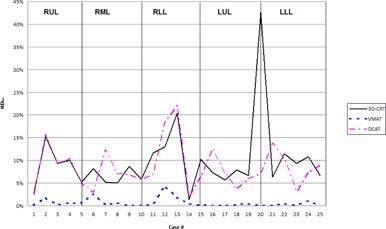
Comparison in the high‐dose location between 3D CRT, VMAT, and DCAT. The high value in Case 20 for 3D CRT is due to a small PTV volume.

**Figure 5:**
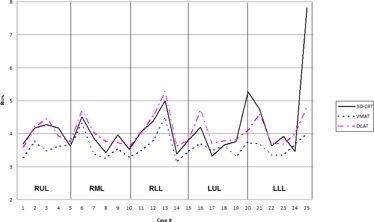
Comparison in the low‐dose volume between 3D CRT, VMAT, and DCAT. The high value in Case 25 for 3D CRT is due to a small PTV volume.

**Figure 6:**
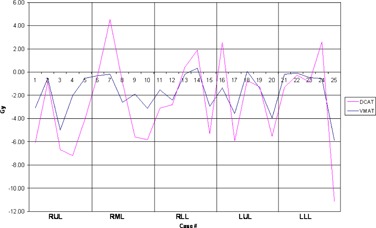
Comparison in the low‐dose location between 3D CRT, VMAT, and DCAT.

High‐dose spillage was analyzed using CI (Fig. 3) and ${\text{HD}}_{\text{loc}}$ indices, while low‐dose spillage was analyzed using $D_{2\text{cm}}$ (Fig. 4) and $R_{50\%}$ (Fig. 5), as suggested by the RTOG. Both VMAT and DCAT were generally able to improve all four indices, providing a more conformal dose distribution to the PTV. When compared with each other, VMAT was superior to DCAT in all four indices. The CI improved in all 25 cases with VMAT, whereas only 11 cases improved with DCAT. Like the CI, ${\text{HD}}_{\text{loc}}$ improved in all 25 cases with VMAT, but only in 14 cases with DCAT. VMAT was unable to improve all cases with $D_{2\text{cm}}$ and $R_{50\%}$. Only one case did not improve $D_{2\text{cm}}$ with VMAT, which was also not met with 3D CRT or DCAT for the same case (Fig. 6). Eight DCAT cases did not improve $D_{2\text{cm}}$, with three of these cases not meeting the respective RTOG standard. Lastly, $R_{50\%}$ improved in 22 cases with VMAT and nine with DCAT. This standard was most difficult to satisfy, with one VMAT case and nine DCAT cases not meeting the respective RTOG standard.

**Table 4:** Summary of mean dosimetric indices for 3D CRT, VMAT, and DCAT treatment plans. Also shown are the VMAT and DCAT plan comparison with respect to 3D CRT plans

	*3D CRT*	*VMAT*	*DCAT*	*VMAT/3D CRT*	*DCAT/3D CRT*
Conformity index	1.16±0.12	0.99±0.02	1.14±0.06	0.85±0.1	0.98±0.1
${\text{HD}}_{\text{loc}}$	9.75±0.08	0.68±0.01	8.15±0.05	0.07±0.0	0.84±0.0
$R_{50\%}$	4.14±0.91	3.57±0.32	4.05±0.48	0.86±0.2	0.98±0.2
$D_{2\text{cm}}$ ^a^	0.56±0.06	0.52±0.06	0.54±0.07	0.93±0.1	0.96±0.2
Lung					
Mean bilateral^a^	0.07±0.03	0.06±0.03	0.07±0.03	0.86±0.6	1.0±0.6
Mean contralateral^a^	0.02±0.02	0.02±0.01	0.03±0.02	1.0±1.1	1.5±1.8
$V_{20}$ (%)	4.02±2.63	3.77±2.9	4.16±3.1	0.94±0.9	1.03±1.0
Ipsilateral brachial plexus^a^	0.04±0.08	0.03±0.06	0.04±0.1	0.75±2.1	1.0±3.2
Proximal bronchial tree^a^	0.32±0.26	0.25±0.24	0.32±0.26	0.78±1.0	1.0±1.1
Esophagus^a^	0.17±0.11	0.13±0.08	0.23±0.09	0.76±0.7	1.35±1.0
Heart^a^	0.33±0.21	0.28±0.24	0.32±0.22	0.85±0.9	0.97±0.9
Spinal cord^a^	0.15±0.10	0.11±0.08	0.21±0.06	0.73±0.7	1.4±1.0
Proximal trachea^a^	0.09±0.14	0.06±0.10	0.1±0.14	0.67±1.5	1.11±2.3
Skin^a^	0.37±0.1	0.37±0.15	0.33±0.15	1.0±0.5	0.89±0.5
Monitor units (MUs)^a^	127±101	179±95	94±63	1.41±1.3	0.74±0.8

a Due to varying prescription dose, individual plan values for any parameter are ratio with respect to the prescription dose.

Results by tumor location by lung lobe were also analyzed (Table 5). VMAT generally provided improvements to all high‐ and low‐dose spillage indices regardless of tumor location. Though DCAT provided improvements to the same indices, when all 25 cases were examined collectively, DCAT was only able to improve all four parameters in the lower left lobe (LLL). The greatest increase to any high‐ or low‐dose spillage indices (DCAT vs 3D CRT) occurred in the ${\text{HD}}_{\text{loc}}$ for the right middle lobe (RML), only increasing by 6.2%. All remaining increases for DCAT remained under 3.5%. For left lung, a good volume is occupied by the heart and ascending arota and other great vessels, leaving very little lung volume. Any change in the dose values in this lung volume is then amplified. For RML and RLL, this is the case as contralateral lung happens to be the left lung. For RUL and LUL, both the volumes of both the contralateral lungs are comparable and, hence, RUL plans behave very close to LUL plans.

**Table 5:** Comparison of mean dosimetric indices of VMAT and DCAT treatment plans with respect to 3D CRT plans

	*Right Upper Lobe (RUL)*		*Right Middle Lobe (RML)*		*Right Lower Lobe (RLL)*		*Left Upper Lobe (LUL)*		*Left Lower Lobe (LLL)*	
	*VMAT/3D*	*DCAT/3D*	*VMAT/3D*	*DCAT/3D*	*VMAT/3D*	*DCAT/3D*	*VMAT/3D*	*DCAT/3D*	*VMAT/3D*	*DCAT/3D*
	*CRT*	*CRT*	*CRT*	*CRT*	*CRT*	*CRT*	*CRT*	*CRT*	*CRT*	*CRT*
Conformity index	0.90±0.04	1.02±0.07	0.88±0.03	1.01±0.05	0.86±0.08	1.01±0.11	0.81±0.15	0.92±0.18	0.82±0.07	0.98±0.10
${\text{HD}}_{\text{loc}}$	0.07±0.10	0.93±0.92	0.11±0.17	1.06±0.57	0.13±0.17	0.99±0.96	0.02±0.02	0.47±0.58	0.05±0.05	0.98±0.51
$R_{50\%}$	0.88±0.54	1.00±0.66	0.92±0.30	1.03±0.32	0.89±0.60	1.03±0.71	0.89±0.30	0.99±0.32	0.79±0.32	0.91±0.35
$D_{2\text{cm}}$ ^a^	0.90±0.11	0.90±0.14	0.95±0.19	1.00±0.17	0.94±0.08	0.99±0.10	0.92±0.20	0.99±0.22	0.95±0.17	0.97±0.20
Lung Mean bilateral^a^	0.92±0.51	1.09±0.61	0.75±0.37	0.85±0.42	0.94±0.51	1.06±0.55	0.86±0.64	0.98±0.75	0.93±0.86	1.11±0.99
Lung Mean contralateral^a^	0.92±0.64	3.25±1.70	1.53±0.56	2.41±0.94	1.45±0.73	1.70±0.57	0.74±0.97	1.19±1.52	0.71±0.97	1.25±1.69
$V_{20}$(%)	0.94±0.67	1.00±0.67	0.97±0.43	1.10±0.46	0.88±0.87	1.00±0.97	0.91±0.83	0.98±0.89	0.97±1.79	1.05±1.90
Ipsilateral brachial plexus^a^	0.45±0.97	0.46±0.94	0.80±1.46	0.96±1.73	0.99±1.08	1.21±1.26	0.92±1.66	1.31±2.52	0.24±0.54	0.28±0.63
Proximal bronchial tree^a^	0.82±0.79	0.93±0.82	0.77±0.83	1.08±1.12	0.74±0.71	1.16±1.19	0.88±1.34	0.93±1.29	0.70±0.73	1.12±1.21
Esophagus^a^	0.74±0.31	1.85±0.96	0.84±0.36	1.66±0.62	0.76±0.57	1.22±0.79	0.76±0.58	1.17±0.76	0.68±0.37	1.23±0.47
Heart^a^	0.71±0.70	0.86±0.76	0.91±0.70	1.16±0.75	0.72±0.31	0.87±0.34	0.91±1.12	0.92±1.06	0.89±0.77	1.04±0.84
Spinal cord^a^	0.79±0.64	1.45±0.91	0.65±0.30	2.56±0.88	0.81±0.75	1.35±0.88	0.69±0.62	0.98±0.66	0.70±0.51	1.46±0.79
Proximal trachea^a^	0.92±1.06	1.60±1.80	0.34±0.55	0.40±0.63	0.97±0.70	1.16±0.82	0.69±0.64	1.11±0.88	0.38±0.69	0.65±1.31

a Due to varying prescription dose, values represent ratio of the studied parameter to prescription dose.

**Figure 7:**
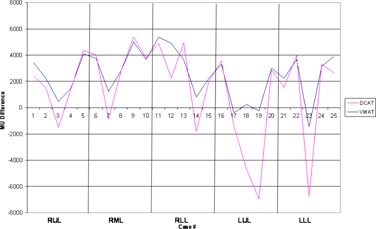
Comparison in the monitor units delivered between 3D CRT, VMAT, and DCAT.

An increase in MUs of 41% occurred with VMAT, while DCAT improved by 26% (Fig. 7). When $\text{MU}/D_{P}$ for each case was normalized to deliver 20 Gy for comparison purposes, the average MUs required for delivery was 2540 ± 2020 MU for 3D CRT, 3580 ± 1900 MU for VMAT, and 1880 ± 1260 MU for DCAT. Only two cases improved with VMAT, while 16 cases improved with DCAT.

## IV. DISCUSSION

A challenge of SBRT is to keep the dose surrounding nearby OARs to a minimum. With respect to this study, no one planning technique was found superior in all aspects with respect to gold standard 3D CRT plans; however, VMAT was able to provide advantages to both 3D CRT and DCAT for a majority of respective RTOG indices.

Both VMAT and DCAT were able to provide a more conformal dose to the PTV, but VMAT was able to achieve better results than DCAT. VMAT has been previously reported to achieve superior conformal plans when compared to 3D CRT and DCAT.^(12)^ The rotating arcs associated with VMAT and DCAT are more effective at conforming dose to spherical PTVs than multiple static beams. When subarcs were used in DCAT plans, dose surrounding the PTV became less conformal as the field weightings were optimized. As a result, high‐ and low‐dose spillage indices increased in value. DCAT planning requires a delicate balance of achieving OAR constraints while not violating high‐ and low‐dose spillage indices. When OARs constraints could not be met, it was due to the OAR lying in close proximity to the PTV.

A concern with the use of intensity‐modulated radiotherapy (IMRT) and VMAT with SBRT lung cancer is whether the motion of the tumor leads to significant dosing discrepancies, especially while delivering a high‐dose per fraction. As the MLCs move across the field, individual leaves may cover part of the target during treatment delivery. Any target movement will result in the dose not being delivered as planned. The interplay between MLC motion, jaw movement, gantry rotation, and target motion during free‐breathing treatments with VMAT is complex when compared to an open field like 3D CRT. In a study of ten patients, Rao et al.^(13)^ found the interplay effect to have a limited impact on target dose distribution with IMRT and VMAT SBRT, where few fractions are delivered. Conventional treatment schemes, where 2 Gy was delivered per fraction, resulted in a larger impact on dose distribution to the target per fraction. This effect was diminished over the course of many fractions associated with conventional fractionation. DCAT, like 3D CRT, remains immune to the MLC interplay effect.

VMAT provided clear advantages to sparing dose to OARs among all treatment planning techniques. Though DCAT increased dose to most OARs, many increases were less than 5% when compared to 3D CRT (Table 4). OAR performance was also examined by tumor location (Table 5). Again, VMAT provided superior results, though DCAT was able to offer some improvements over 3D CRT. However, no single location was superior in all OARs for DCAT. No improvements were observed in the esophagus or spinal cord for DCAT when examined by tumor location. A clear relationship between reducing OAR doses and DCAT could not be established as a function of tumor location in different lobes. A limitation of this study is only five cases were examined per lung lobe. Further study on the role of tumor location is recommended to establish more conclusive results.

The increase in volume of normal tissue receiving a low radiation dose associated with arc‐based techniques does remain a concern. The potential danger in increased low‐dose volumes is the risk of secondary malignancies in these tissues.^(14)^ Shorter treatment times and improved OAR sparing results in larger low radiation dose regions in normal tissues, more commonly in the contralateral lung.^(15)^ Ong et al.^(12)^ reported an increase in bilateral $V_{20}$ from 4.9% to 5.4% in a study of 18 patients comparing VMAT and 3D CRT. These findings contradict our findings in which $V_{20}$ decreased by 5.4% when comparing VMAT with 3D CRT. This discrepancy may be explained by the fact that only half of the patients in the Ong study used VMAT planning objectives for the contralateral lung, whereas all patients in our study used such constraints. The use of partial arcs to avoid the contralateral lung reduces its dose,^(4)^ as we did for some patients (n = 17) in our study. The risk of pneumonitis also remains a concern with arc‐based techniques. Palma et al.^(15)^ examined radiological lesions of 25 patients treated with VMAT and 50 patients with 3D CRT after receiving SBRT. It was concluded that VMAT and 3D CRT results in similarly low rates of clinical pneumonitis and similar severity and patterns of radiological changes in the lungs.

The accuracy of patient setup for SBRT, regardless of the treatment technique, is crucial due to the high dose delivered per fraction and the surrounding steep dose gradients. Morales‐Paliza et al.^(16)^ evaluated the variation in tumor dose coverage between IMRT and DCAT when the isocenter was shifted by 2 mm from the original location. The DVHs between the shifted and nonshifted plans were compared, and DCAT showed less sensitivity than the corresponding IMRT dose. While steep dose gradients associated with IMRT and VMAT can be great advantages, it can also result in a deviation from the prescribed dose due to set up errors, if not corrected.

Delivery of such ablative doses depends on our ability to target the tumor precisely before and during the treatment. This is usually achieved using image‐guidance radiotherapy (IGRT), which adds to the total treatment time. Adjustments to patient position are made if required, reducing the amount of setup error during treatment. Prolonged delivery times can be difficult for patients to tolerate, especially in the presence of abdominal compression plates. Faster delivery techniques have the potential to increase patient comfort while reducing the amount of patient motion during treatment, improving treatment accuracy. Shorter delivery times are thought to be more effective due to the decreased time available for repair of radiation‐induced DNA damage.^17^, ^18^ Faster SBRT delivery also allows for a more effective use of the treatment machine and departmental resources.

SBRT requires more time to deliver treatments than conventional radiotherapy due to the complex initial setup and image guidance using integrated imaging. Initial patient setup for all three treatment techniques would be identical due to the use of a single isocenter, requiring the same amount of time to verify patient position using integrated imaging. Any improvements in delivery times would be seen in the treatment following patient setup. If using 3D CRT, gantry, collimator, and couch positions are changed after each field has been delivered, lengthening the total treatment time significantly. Tumor position has been shown to shift for treatment times exceeding 15 min.^(19)^ Further imaging can correct such motion, but would lead to lengthened delivery times. Studies have reported a reduction in delivery time with VMAT compared to 3D CRT.^4^, ^12^ DCAT has also been shown to provide reduced treatment times when compared to IMRT among multiple target sites.^(16)^ Ross et al.^(20)^ observed a reduction in delivery time by 42% when using DCAT compared to 3D CRT. VMAT and DCAT has been shown to shorten both treatment planning and delivery times for lung SBRT, leading to a more comfortable experience for the patient. Improved patient comfort can reduce target motion due to discomfort during treatment and reduce the need for additional integrated imaging necessary for longer treatments.

MUs required for treatment delivery increased by 41% with VMAT, while DCAT provided an improvement of 26% over 3D CRT. Though MUs required to delivery VMAT plans increased, Ong et al.^(12)^ reported shorter treatment times when compared to 3D CRT or IMRT. In a separate study, a decrease in MUs required for DCAT was also observed when compared to IMRT among multiple target sites.^(16)^ The reduction in MUs in DCAT over VMAT in this study makes DCAT an appealing alternative to VMAT, especially in facilities that do not have VMAT capabilities. While treatment planning systems and linear accelerators with VMAT capabilities are comparatively expensive, DCAT provides a cost‐effective method to provide a comparable dose delivery for SBRT lung cases as it uses standard computation algorithms, as well as simple linear accelerators. Besides, DCAT is not affected by MLC interplay effect and is less susceptible to slight setup errors or smaller changes in breathing patterns. All these provide better confidence in the dose delivery accuracy with DCAT. It provides the fastest delivery, due to least amount of MUs among the three modalities, and also helps in reducing intrafraction motion and improving patients' comfort. DCAT plans, however, are manually optimized, using forward‐planning techniques and, hence, depend on the proficiency and experience of the planner.

We have used analytic anisotropic algorithm (AAA) for dose calculations, which is a RTOG‐acceptable algorithm for dose computations for RTOG lung protocols. Several papers have pointed out the limitation of AAA in the presence of low‐density heterogeneity.^21^, ^22^, ^23^, ^24^, ^25^ Recently, a number of investigators have recommended the use of Acuros XB, a new dose calculation algorithm available in Eclipse, for dose calculations in lung treatment plans.^21^, ^22^, ^23^, ^24^, ^25^ We intend to come out with a follow‐up paper in future, describing our results using this algorithm.

Recent studies examining the potential dosimetric benefits of VMAT SBRT with flattening filter‐free (FFF) beams in the treatment of early‐stage NSCLC show promising results.^26^, ^27^, ^28^, ^29^, ^30^ Navarria et al.^(26)^ saw a decrease in beam‐on time from about 8 min to 2 min when comparing FFF VMAT SBRT with four to six conformal arcs in a study of 132 patients. The local control rate at one year was 100% for patients treated with FFF VMAT SBRT. Ong et al.^(27)^ retrospectively compared SBRT VMAT plans in both FFF and flattening filter mode. Plan conformality, PTV coverage, and OAR sparing were comparable for both techniques, though FFF plans required an increase in MUs of about 8%, though treatment times for FFF beams reduced delivery times significantly. Both studies utilized 10 MV photon FFF beams with a dose rate of 2400 MU/min. Even though FFF fields were not used for either VMAT or DCAT in this study, it is expected that the higher dose rates offered by them will further reduce the treatment delivery times.

One of the advantages often overlooked in comparing 3D CRT plans with VMAT and DCAT plans is the potential benefit if the patient returns for future treatment due to another new or recurrent lesion in the thorax. An overlap between the old and new regimens is not preferred. Since 3D CRT plans typically use noncoplanar beams, designing another plan with the same planning technique without any overlap becomes much more challenging and nonintuitive. However, VMAT and DCAT plans are generally coplanar in nature and, hence, it is easier to predict any overlap with earlier treatments, making it much easier to plan with more confidence in avoiding overlap, not to mention the strength of inverse optimization in generating a new plan. Considering the fact that SBRT is a relatively new technique, clinical data in handling any form of future plan overlap are still being explored. Due to excellent results with SBRT, we are seeing a good number of such patients requiring reirradiation to the chest for new or recurrent tumors.

## V. CONCLUSIONS

Based on the dosimetric indices compared in this study, VMAT provides a superior treatment plan to 3D CRT and DCAT in the treatment of medically inoperable early‐stage NSCLC using SBRT. Though VMAT and DCAT improved dose distributions to the PTV, VMAT provided more dose sparing to OARs. DCAT should still remain as an alternative to 3D CRT in facilities that do not have VMAT or IMRT capabilities, and offers its own advantages.
